# Recent Developments on Coumarin Hybrids as Antimicrobial Agents

**DOI:** 10.3390/antibiotics14121226

**Published:** 2025-12-05

**Authors:** Sijongesonke Peter, Lunga Linda Sibali

**Affiliations:** 1 Department of Chemical and Earth Sciences, Faculty of Science and Agriculture, University of Fort Hare, Alice 5700, South Africa; 2 Office of the Dean, Faculty of Science and Agriculture, University of Fort Hare, Alice 5700, South Africa

**Keywords:** antibiotics, coumarin, microbial infections, hybrids, drug resistance, antimicrobials

## Abstract

**Introduction**: Globally, microbial infections are projected to be among the leading causes of death by 2050 due to rising drug resistance. Antimicrobials are vital for treating both animals and humans worldwide. However, their overuse and misuse accelerate drug resistance, posing a serious threat to public health. Coumarin is a naturally occurring compound contributing health-beneficial features in drug discovery. Its high solubility in organic solvents, high bioavailability, simple structure, low toxicity, and low molecular weight make it an ideal candidate for combining with other pharmacophores to develop new therapeutic agents. This compound exhibits several biological activities, including antimicrobial, anticancer, anti-inflammatory, antidiabetic, neuroprotective, and anticoagulant effects, motivating medicinal researchers to hybridize it with other compounds to enhance its pharmacological efficacy. Hybridization of different pharmacophores via suitable linkers, including cleavable and non-cleavable ones, is a promising approach in drug development, resulting in new therapeutics with improved biological activity. Therefore, the hybridization of coumarin with other pharmacophores has become an interesting paradigm for medicinal scientists. Aim: This review aims to summarize the existing scientific literature on coumarin-based hybrid compounds with antimicrobial capabilities and discuss the structure–activity relationship (SAR) of these hybrids to potentially guide future research on and development of coumarin-based drugs for microbial treatment. **Material and Methods**: The review focuses on open-access literature about coumarin hybrid drugs available through searching tools such as Google, Google Scholar, ScienceDirect, and Scopus, published from 2024 to 2025. **Results**: Coumarin hybrids exhibit promising antimicrobial activity, particularly against *S. aureus* and *C. albicans*. The SAR reveals that halogenation, bulky aromatics, nitro, and hydroxyl groups enhance the interaction of the coumarin rings with amino acid residues. **Conclusions**: The reported coumarin hybrids showed a promising antimicrobial activity, with structural modifications influencing their activity. Hence, more studies, including more pre-clinical and clinical evaluations, are recommended for these hybrid compounds.

## 1. Introduction

Globally, antimicrobial resistance is a major challenge contributing to the increasing number of microbial infections [1,2,3]. This antimicrobial resistance is triggered by the mutating genes, resulting in bacterial, fungal, parasite, and viral species being insusceptible to currently used antimicrobial drugs [3,4]. Among these infections, bacterial infections are contributing significantly to the rise in cases, with fungal pathogens and viruses also being responsible for many hospitalizations [5,6]. Consequently, a rise in the number of hospitalizations, cases, and deaths due to microbial infections is expected by the year 2050 [7]. The prevalence of these infections even threatens the treatment of other communicable and non-communicable diseases, placing a strain on the public health system [4,6]. Therefore, there is a pressing need for more alternative drugs to treat microbial infections.

Natural-originated drugs, including drugs extracted from plants, show promising biological effects and offer more health benefits [8,9]. Therefore, medicinal researchers are showing an increasing propensity to opt for plant-based drugs to treat modern diseases. As a result, most of the currently used therapeutic agents originate from plants [8,9]. However, the development of new drugs has slowed, with only a few reaching the market [10,11]. Hence, more innovative therapeutic agents are being researched to advance the drug discovery industry. Thus, coumarins are among the plant-based molecules gaining momentum in medicinal chemistry.

Coumarins are polyphenolic plant-based molecules that were first extracted from the *Dipteryx odorata Willd* plant in the early 1820s [12]. They are abundant in the leaves, seeds, and roots of many plants, including the *Rutaceae* and *Apiaceae* families [12,13,14,15]. These compounds and their derivatives have significantly contributed to drug discovery, as they possess several biological activities, including anticancer, antimicrobial, anti-inflammatory, and anticoagulant effects [12,13,14,15]. Additionally, coumarins also contribute to the development of cosmetics [12,13]. However, they can trigger hepatoxicity at high doses [12,16]. Hence, alternative strategies to enhance their biological efficacy are urgently needed. As a result, several researchers hybridize these bioactive molecules with other pharmacophores to enhance their biological efficacy.

The structural modification of the compounds influences their biological efficacy [12,16]. Thus, several compounds are combined via both cleavable and non-cleavable linkers using suitable functional groups to form hybrid drugs [17,18,19]. Hybrid drugs are a cocktail of drugs combined to form a single-entity molecule with a better synergistic effect [17,18,19]. These hybrid drugs are reported to overcome challenges such as drug resistance, toxicity, drug–drug interactions, and side effects associated with the single-entity drugs, polytherapy (PT), and fixed-dose combination (FDC) therapy [17,18,19,20]. Thus, the hybrid strategy is complemented by a drug repurposing strategy, with both exhibiting cost and time efficiency. Drug repurposing/reprofiling refers to the use of known drugs with known pharmacokinetics and pharmacodynamics for different biological activities [21,22,23]. Hence, drug reprofiling through hybridization is an interesting approach and is attracting increasing amounts of attention from medicinal scientists. This review surveys the literature, aiming to document a compilation of recently reported coumarin hybrid compounds with antimicrobial activity. This review provides an update on the contribution of coumarin in drug discovery and possibly paves the way forward for future research and improvements to enhance its biological efficacy. The structural activity relationships and in silico, in vitro, and in vivo behavior of these compounds will be thoroughly analyzed in this review.

## 2. Significance of Plant-Based Compounds to Drug Discovery

**Figure 1:**
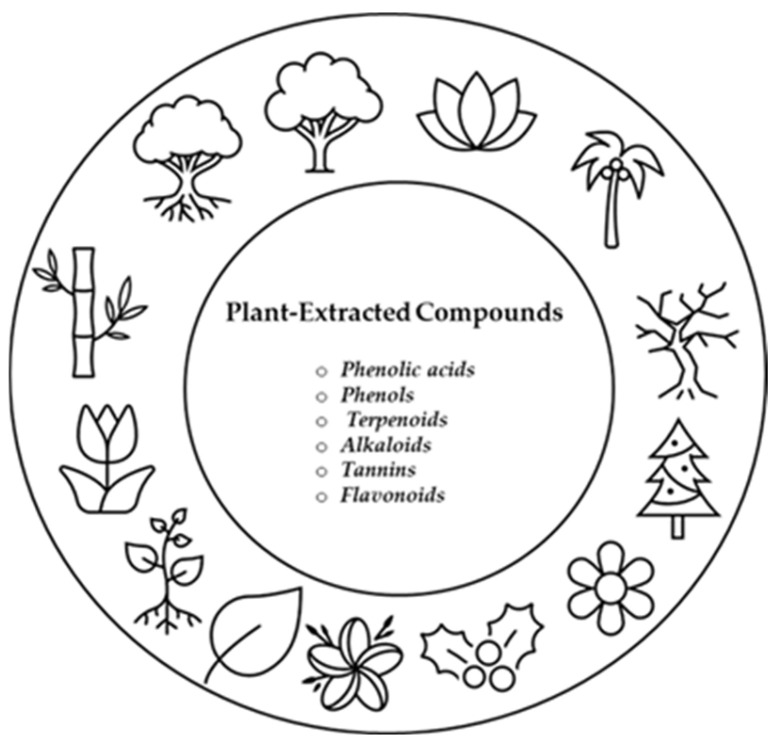
Examples of plant-based compounds.

The use of plant-extracted compounds (displayed in Figure 1) to develop phytopharmaceuticals is an interesting paradigm that has attracted interest from many medicinal scientists. Traditional medicines such as antimicrobials support about 85–90 percent of the world’s population in terms of treating existing diseases [24,25,26,27]. Due to their cost-effectiveness, health benefits, and tolerable toxicity, they offer quick access and affordable medication as single drugs and in combination therapy [9,25,28]. Additionally, plant-origin compounds are utilized as precursors to develop new therapeutic agents. Thus, some plant-origin compounds such as artemisinin, quinine, codeine, etc., are involved in clinical trials [25,28]. These promising clinical applications of plant-origin drugs motivate the pharmaceutical industry to explore their use in the development of new therapeutic agents for various therapeutic applications. Hence, this review focuses on hybrid compounds containing coumarins with antimicrobial activity.

## 3. Coumarins

**Figure 2:**
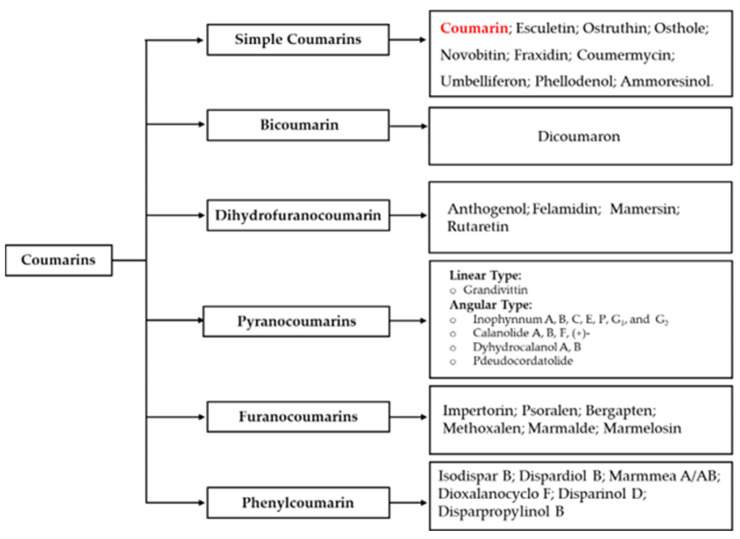
Classification of coumarins.

Coumarins are a group of naturally occurring compounds that are commonly extracted from plant roots, leaves, and seeds of the benzopyrone compounds-containing plant family [29]. More than 1800 coumarins and their derivatives have been identified naturally and synthetically, and they are categorized into six different classes based on their complexity, simplicity, and chemical diversity [29,30,31,32,33]. These six classes include simple coumarins, bicoumarin, dihydrofuranocoumarin, pyranocoumarins, furanocoumarins, and phenylcoumarin, as displayed in Figure 2 [29,30,31,32,33]. They display several advantages, including a simple chemical structure, low molecular weight, limited side effects, simple dissolution in organic solvents, high bioavailability, low resistance, and a wide range of biological activities [34]. Additionally, they are rich in electrons, making them easily react with different molecules, which is the factor behind their pharmacological efficacy [12,29,32,35]. Hence, they are attracting attention from scientists for structural modifications and hybridization.

**Figure 3:**
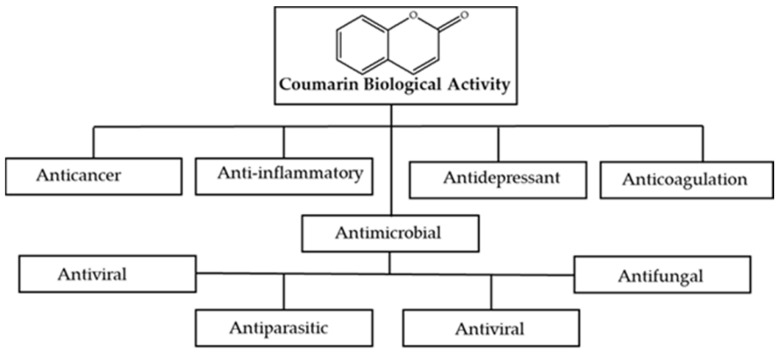
Pharmacological properties of coumarin.

As depicted in Figure 3, coumarins have been proven to be potential therapeutic agents, exhibiting antidepressant, anti-inflammatory [36], antiviral [37], anticoagulant [38], and anti-proliferative [39] as well as antimicrobial activities. The benzopyrone moiety is reported to contribute to their biological efficacy, with structural modifications reported to enhance the biological efficacy of these compounds [33,36,37,38,39]. For instance, the chloro-substituted coumarins result in a significant antifungal agent, with the presence of a hydroxyl group at position 7 of coumarins showing antifungal and antibiotic properties [40]. The antimicrobial efficacy is triggered by blocking quorum-sensing signaling systems and inhibiting the growth of pathogens, resulting in their death [41]. Specifically, coumarins’ antibacterial potency is primarily attributable to their capability to bind to the B subunit of bacterial DNA gyrase and prevent DNA secondary structures by inhibiting ATPase at cellular and molecular levels, respectively [42]. Additionally, these compounds can also inhibit topoisomerase II and bind to the nucleotide-binding site of gyrase B, leading to an antibacterial effect. Notably, coumarins can act against *E. coli* by hindering *E. coli* FtsZ, resulting in inhibition of bacterial cell division [16].

## 4. Material and Methods

The literature documented in this review article was searched using searching tools such as Google Scholar, Scopus, ScienceDirect, and Google using keywords such as coumarins, coumarin hybrids with antibacterial activity, coumarin derivatives, and coumarin antimicrobial activity. Only open access articles published between the years 2024 and 2025 were downloaded and documented in this review article. There are recent review articles documenting coumarin hybrids’ various biological activities. Hence, the authors focused on the years 2024 to 2025 to expose the progress that has been made in establishing coumarin hybrids as antimicrobial agents. The authors used ChemDraw^®^ Ultra (version 8.0) software to redraw the structure of the antimicrobial coumarin hybrids most frequently extracted from the literature.

## 5. Coumarin Hybrid Compounds with Antimicrobial Activity

**Figure 4:**
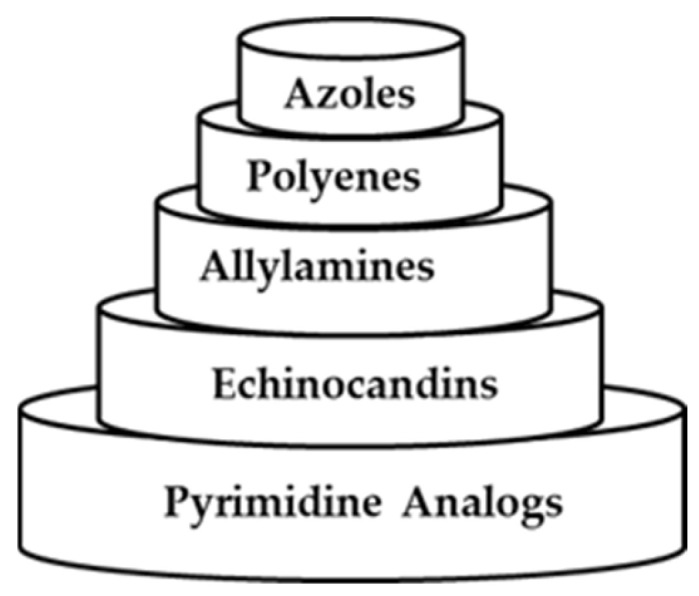
Classes of antifungal drugs.

Globally, fungal infections are causing a strain on public health systems, as more cases and fatalities are being reported [43,44]. This is due to the limited class of both synthetic and natural antifungal drugs (depicted in Figure 4) available on the market, contributing to the increasing number of fungal infection casualties, as fungal pathogens such as *Candida*, *Aspergillus*, and *Cryptococcus genera* develop defensive mechanisms against these antifungal drugs [43,44,45,46]. Additionally, narrow therapeutic indices and poor oral bioavailability also hamper the efficacy of the currently used drugs [43].

On the one hand, bacterial infections also contribute to the increasing number of deaths, cases, and hospitalizations, due to the failure of the currently employed therapeutic agents [47,48]. Antibiotic resistance is a major contributor to the failure of the commercially available drugs, and this has become a concern for medicinal scientists, who are seeking an alternative approach to overcome the challenges associated with currently used drugs [49,50,51]. Hybridization is among the most beneficial structural modification strategies gaining momentum in the drug discovery industry [49]. The drugs developed using this approach displayed a series of advantages, including reduced drug–drug interactions, improved pharmacokinetic properties, reduced toxicity, and improved patient compliance [50,51]. Hence, scientists are hybridizing different pharmacophores with coumarin, resulting in new antimicrobial agents with enhanced therapeutic efficacy.

**Figure 5:**
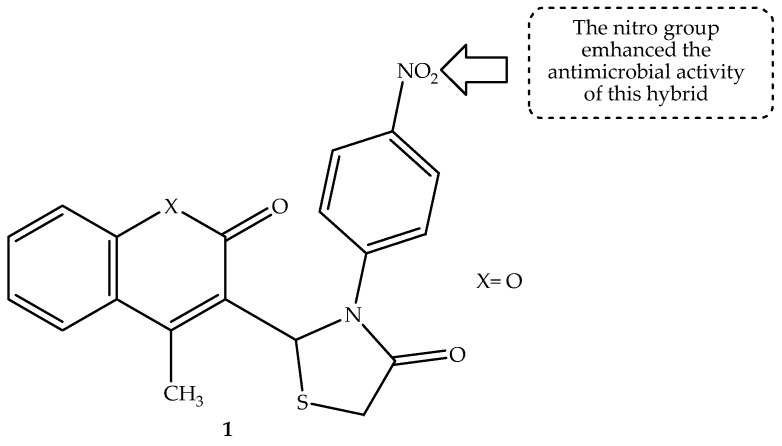
Chemical structure of coumarin-thiazolidinone hybrid **1**.

The use of the heterocyclic compound to develop new compounds has gained momentum. Thus, Abbas et al. [52] reported that 60% of the most successful pharmaceuticals available on the market contain heterocyclic scaffolds. Hence, coumarins are combined with other scaffolds to develop drugs for microbial infections. Farhan and co-partners synthesized new antimicrobial agents via a combination of 4-thiazolidinone and a coumarin scaffold [53]. The naturally occurring scaffold (coumarin) and the bioactive motif (4-thiazolidinone) are showered with several biological applications, and they possess antifungal, anticancer, antibacterial, anti-HIV, and anti-inflammatory activities, making them a powerful match to develop new hybrid compounds with antimicrobial activity [53]. The preliminary studies against antibacterial strains and human antifungal strains showed compound **1** (displayed in Figure 5) to be the most antimicrobial active compound compared to its counterparts. However, all the synthesized hybrids showed inferior antimicrobial properties with inhibition zones (displayed in Table 1) between 8 and 17 mm compared to 20 and 26 mm for the reference drugs. Notably, the type of substituents on the hybrids influenced their antimicrobial effect, with the nitro group-containing hybrids exhibiting a more pronounced antimicrobial effect compared to those containing bromo, chloro, and methyl groups [53]. Therefore, more evaluations, including clinical studies, are paramount to validate the outcomes of this study.

**Figure 6:**
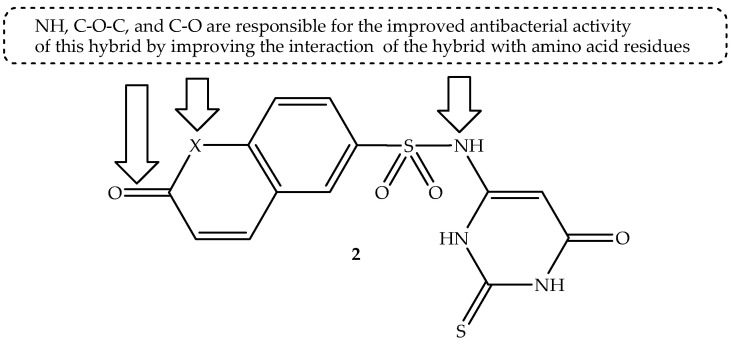
Chemical structure of coumarin-sulfonamide hybrid **2**.

Abo-Salem et al. [54] synthesized a generation of coumarin-sulfonamide hybrids through a combination of the coumarin scaffold with sulfa drugs and amino-heterocyclic compounds. The antimicrobial activity of these hybrids was evaluated in vitro against two antibacterial strains (*S. aureus* and *E. coli*) and human antifungal strains (*C. albicans* and *A. niger)* [54]. The compounds behaved differently against antimicrobial pathogens, with hybrid **2** (depicted in Figure 6) showing a better inhibition zone (28–39 mm) against all the antimicrobial strains, in contrast to other hybrids (0–26 mm) and neomycin (0–28 mm), as depicted in Table 2 [54]. This was validated by the MIC results, as this compound exhibited four-fold more antimicrobial activity with MIC values of 4.88 and 9.76 µg/mL against *S. aureus* and *C. albicans*, compared to 19.53 and 39.06 μg/mL of neomycin, respectively [54]. Consequently, this hybrid depicted a greater ability to inhibit DNA gyrase compared to other tested compounds with an IC_50_ value of 1.793 μg/mL, which is close to that of a well-known DNA gyrase inhibitor (novobiocin) with an IC_50_ value of 1.425 µg/mL [54].

In silico molecular docking studies revealed that compound **2** displayed a comparable binding energy with novobiocin against DNA gyrase B targets, showing interaction with DNA gyrase B amino acid residues by forming the hydrogen bond via NH, SO_2,_ and CO groups. It further interacted with the amino acid residues via alkyl, pi–sigma, and pi–alkyl interactions through benzene, pyrone, and cyclohexane. Hence, the SAR illustrated that the NH of the pyridine ring, C-O-C, and C-O of the pyrone ring were paramount in suppressing the DNA gyrase, leading to cell death [54]. Moreover, the in silico ADME studies revealed that hybrid **2** showed a good bioavailability score of 0.55, water solubility (LogS = −2.56), and drug likeness with no Lipinski’s rule violation, except one minor violation of TPSA, which was greater than 140 Å^2^. In essence, Hybrid **2** displayed good antimicrobial ability, and it can be recommended as a potent antimicrobial candidate among the synthesized hybrids [54].

**Figure 7:**
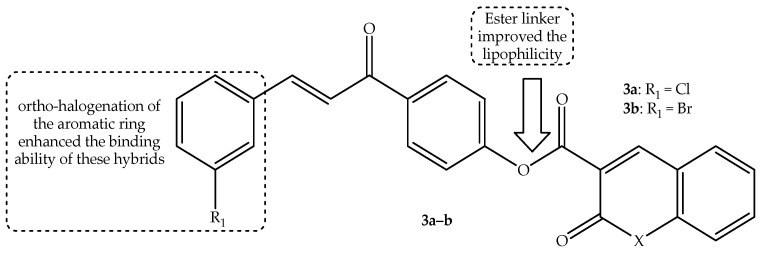
Chemical structure of coumarin–chalcone ester-linked hybrid **3a**–**b**.

The pharmacological effect of two compounds of natural origin, coumarin and chalcones, including their derivatives, led to Ngaini et al. [55] to hybridize these two scaffolds to enhance their synergistic effect. The ester-linked halogenated hybrids were synthesized and tested against two bacterial strains, *E. coli* and *P. aeruginosa*, in vitro. The hybridization of these two pharmacophores resulted in more antibacterial activity in hybrids in comparison to their parent compounds [55]. Notably, the compounds exhibited marginally more antibacterial activity against *P. aeruginosa* compared to *E. coli*, and this was attributed to the thick bacterial cell walls and outer membrane layers of the Gram-negative *E. coli*. Hence, hybrid **3a** and **3b**, shown in Figure 7, were the most antibacterial effective agents with an inhibition zone (shown in Table 3) of 12 mm and 13 mm against *E. coli* and *P. aeruginosa*, respectively [55].

It was noted that the introduction of either a chloro or bromo group in the ortho position of the chalcone moiety resulted in enhanced antibacterial effect, illustrating the ortho position as an ideal site of halogenation, as they displayed good interaction and binding capability with the protein receptors [55]. Additionally, the ester linkage also contributed to the enhanced lipophilicity, which activated the antibacterial mechanism against *P. aeruginosa*, with bromine further contributing to the increased lipophilicity of the hybrids, promoting hydrophobic interactions between the cell walls of *E. coli* [55]. Therefore, in silico molecular docking studies were conducted to validate these findings. The docking results confirmed that hybrid **3a** and **3b** are potential antibacterial candidates with good binding interactions with protein receptors via the hydrogen bonding, aromatic, Van der Waal, and electrostatic interactions using the ketonic moiety of coumarin, the unsaturated ketonic moiety of chalcone, ortho-chlorine, and ortho-bromine with binding scores of −10.1 kcal/mol and −9.1 kcal/mol, respectively. In essence, the aromatic rings, C=O, HC=CH, and C-O, were responsible for the enhanced activity of these hybrids [55].

Moreover, in silico ADMET predictions collaborated with the docking studies, as these two hybrids displayed good drug likeness with no violation of Lipinski’s rule and a good bioavailability score of 0.55. Additionally, the compounds displayed the ability to pass the blood–brain barrier, illustrating that they could be beneficial for the treatment of bacterial-related central nervous system disorders. Furthermore, the drugs displayed higher total clearance in the body, with compound **3a** showing less toxicity. This illustrates that hybrid **3a** was the most satisfactory antibacterial candidate in vitro and in silico [55]. Therefore, more clinical trials are recommended for this hybrid.

**Figure 8:**
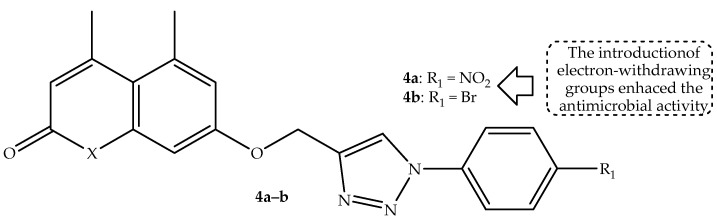
Chemical structures of 1,2,3-triazole-linked coumarin hybrid **4a**–**b.**

Dhawan et al. [56] synthesized a series of 1,2,3-triazole-linked coumarin hybrids through a combination of coumarin and azide-substituted moieties via a copper-assisted 1,3-dipolar cycloaddition reaction and evaluated their antimicrobial activity against two fungal and five bacterial strains. Hybrid **4a** (displayed in Figure 8) with a nitro group at the para-position of the aromatic ring exhibited the most antibacterial activity among the synthesized hybrids, displaying zone inhibition (displayed in Table 4) of 14.8 mm and 15.43 mm against *P. aeruginosa* and *S. aureus*, respectively. Furthermore, hybrid **4b** (depicted in Figure 8) displayed a better antifungal effect against *C. albicans* with a zone of inhibition of 10.43 mm [56]. Notably, the SAR highlighted that mono-substituted hybrids with the most electron-withdrawing groups exhibited better antimicrobial activity compared to those with electron-donating groups. Additionally, the alkyl chain length and aromaticity showed no significant influence on the antimicrobial activity of the hybrids. Moreover, in silico DFT predictions displayed that these hybrids are reactive and stable towards both nucleophilic and electrophilic entities [56]. Hence, further studies are recommended to reinforce these findings.

**Figure 9:**
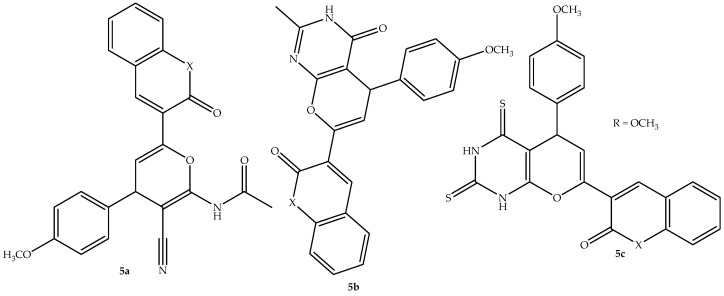
Chemical structure of coumarin–chromene-based hybrid **5a**–**c.**

Interestingly, Fayed et al. [57] synthesized coumarin–chromene-based hybrid compounds via different synthetic routes and evaluated their antimicrobial activity against different bacterial and fungal strains. The hybrids behaved differently against the bacterial strains, with **5a**–**c** (depicted in Figure 9) exhibiting a significant antibacterial effect, which was comparable to the reference drugs (ciprofloxacin and cephalexin), on *P. aeruginosa* and *E. coli* bacterial strains with MIC values (shown in Table 5) between 0.25 and 1 μg/mL, with other synthesized hybrids displaying moderate or weak antibacterial activity with MIC values that were more than 1 μg/mL against various bacterial strains. Additionally, none of the hybrids has displayed a significant activity against the antifungal strain (*C. albicans*), in vitro. Thus, the antibiofilm, DNA gyrase inhibition ability, and in silico evaluation were conducted for hybrid **5a**–**c** to supplement the in vitro findings.

The SAR observations demonstrated that the coumarin moiety was responsible for the potent antibacterial activity of these hybrids, with the incorporation of different substituents on the side chains of the hybrids negatively influencing the biological effect of the hybrids against the used antimicrobial strains [57]. These three hybrids exhibited a noticeable reduction in bacterial biofilm mass compared to the positive control, novobiocin. Furthermore, among the three hybrids, compound **5a** displayed a significant ability to inhibit DNA gyrase supercoiling against *E. coli* genes with an IC_50_ value of 5.052 μM, which was comparable to that of novobiocin (IC_50_ = 3.306 μM).

This was supplemented by the docking studies, as hybrid **5a** displayed a good binding energy with the DNA gyrase via hydrogen bonding with a binding score (−19.49 kcal/mol) comparable to that of novobiocin (−20.78 kcal/mol) [57]. Hence, this hybrid was regarded as the compound that exhibited the most antibacterial activity among the synthesized hybrids. The in silico ADMET predictions confirmed this compound as a potential therapeutic candidate from the series, as it displayed no violation of drug likeness rules, a good bioavailability score (0.55), no ability to pass the BBB, and it is not a substrate of P-gp. Additionally, this hybrid showed no toxicity threats except for a compromising factor of hepatotoxicity [57]. Therefore, more clinical trials are recommended for this drug to validate its potential as an antibacterial drug candidate.

**Figure 10:**
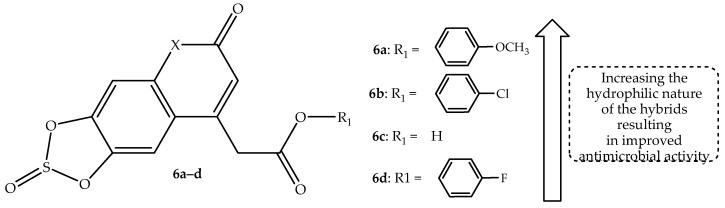
Chemical structure of coumarin-based hybrid **6a–d**.

Zeki et al. [58] synthesize coumarin-based hybrid compounds as potential antimicrobial agents when tested against ten bacterial (four anaerobic and six aerobic Gram-negative), two fungal strains, and three normal microbiota non-pathogenic strains, with ciprofloxacin used as a reference drug. The hybrids exhibited less antibacterial activity compared to ciprofloxacin against aerobic Gram-negative bacterial strains, but better antibacterial activity compared to their parent compounds, with MBC and MIC values ranging between 2 and 29 μg/mL and 2 to 16 μg/mL, respectively [58]. Hybrid **6a**–**b** (shown in Figure 10) with a methoxy and chloro group at the para-position of the phenyl ring, displayed better antibacterial activity against selected bacterial strains compared to their counterparts, as observed on MIC values displayed in Table 6. The SAR showed that the increasing hydrophobic nature of these hybrids influenced their antibacterial activity [58]. Additionally, it was highlighted that increasing the hydrophilic and lipophilic nature of the hybrids, as was the case in compound **6b**, results in a better antibacterial effect, as this improves the ability of a drug to break down the bacterial cell walls. Moreover, the presence of the dioxathiole ring contributed to the improvement of the antibacterial activity of the hybrids [58]. However, a hydrophilic functional group (carboxylic group) in hybrid **6c** compromised the antibacterial activity of that compound. This demonstrates the significance of the phenyl ring in the improved antibacterial effect of these hybrids [58].

Furthermore, three hybrids, **6b**–**d** (depicted in Figure 10), showed significant results against the fungal strains (*C. albicans* and *A. niger*), with superior MFC and MIC values between 1.30 and 2.50 µg/mL and 1.15–1.80 µg/mL, compared to 6–12 µg/mL and 4–8 µg/mL of the reference drug (nystatin), respectively [58]. The antifungal results highlighted the importance of the dioxathiole ring in enhancing the antimicrobial effect of these hybrids. Additionally, varied substituents at the para-position of the phenyl ring influenced the antifungal activity of the hybrid in this hierarchy: COOH < *p*-phenyl-F < *p*-phenyl-Cl < *p*-phenyl-OCH_3_ < *p*-phenyl-CH_3_ < *p*-phenyl-Br < *p*-phenyl-I. The hybrids showed promising results against the normal strains, with compound **6b** displaying remarkable results against *E. coli* non-pathogenic strains in vitro. This illustrates that hybrid **6b** can be a potential candidate to treat microbial infections [58].

The in silico findings displayed the hybrids as good drug candidates. Specifically, they showed P-gp inhibition ability, which is a good feature for improving drug bioavailability. However, its inhibition could result in the development of interactions [58]. Hence, hybrid **6c** (P-gp non-substrate) could be recommended instead of hybrid **6a** (P-gp substrate). Moreover, the drugs displayed no violation of Lipinski’s rule, showing a good drug likeness and promoting oral administration, and the hybrids cannot pass the BBB, displaying no toxic threats to the CNS. In essence, the findings demonstrate that dioxathiole ring-containing coumarin hybrids **6b** and **6c** could be potential antimicrobial agents [58]. Hence, more clinical trials are recommended.

**Figure 11:**
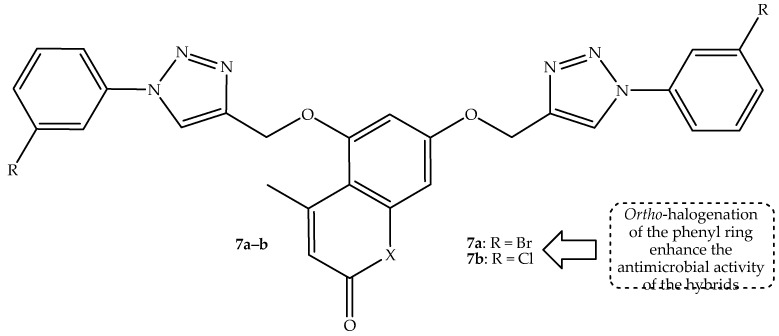
Chemical structure of coumarin–bis-triazole hybrids **7a**–**b**.

Another generation of coumarin–bis-triazole hybrids was synthesized and reported by Dhawan et al. [59]. The compounds contain different alkyl chain lengths between the phenyl rings and the triazole linkers, and they were tested against two fungal (*C. neoformans* and *C. albicans*) and different bacterial (*K. pneumonia*, *P. aeruginosa*, *A. baumannii*, and *E. coli*) strains in vitro. The hybrids displayed disappointing antimicrobial activity, with no significant trend against the used strains. It was *ortho*-halogenated hybrid **7a** (bromo) and **7b** (chloro), shown in Figure 11, with percentage inhibition growth results shown in Table 7, which display better activity among the synthesized compounds against *K. pneumonia* and *C. albicans*, illustrating that bromo and chloro groups can improve the antimicrobial activity of the drugs [59]. This was also reported by Perz et al. [60]. Therefore, further structural modification and in silico ADMET studies are recommended for these two compounds.

**Figure 12:**
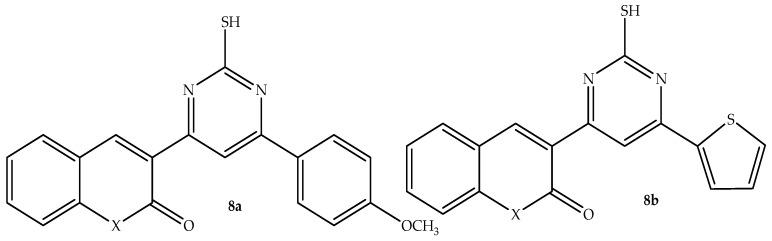
Chemical structure of chalcone–thiourea-linked coumarin hybrids **8a**–**b.**

The contribution of Chalcones to the development of heterocyclic compounds triggered Abd-Elhussain et al. [61] to synthesize chalcone–thiourea-linked coumarin hybrids and evaluate them against different microbial strains to produce potent drugs for microbial infections. The antibacterial findings showed that compound **8a** (depicted in Figure 12) exhibited better antibacterial activity among the synthesized hybrids, with a zone of inhibition (depicted in Table 8) ranging from 10 to 17 mm, compared to 15 to 22 mm of ciprofloxacin against both Gram-negative and Gram-positive bacterial strains. Although all the synthesized compounds showed promising antifungal activity, it was hybrid **8b** (depicted in Figure 12) that showed superior antifungal activity against *C. albicans,* with a zone inhibition of 25 mm compared to 30 mm of fluconazole in vitro [61]. Hence, more studies, including SAR, in silico, and more clinical studies, are needed for these two promising effective antimicrobial hybrids to reinforce these findings.

**Figure 13:**
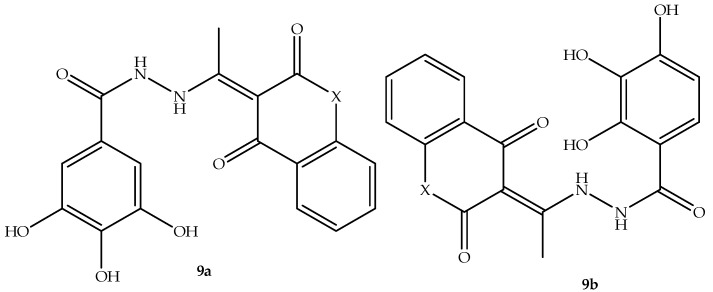
Chemical structures of pyrogallol–coumarin-based hybrids **9a**–**b**.

The progress of coumarin derivatives to clinical trials motivated many medicinal scientists to utilize the coumarin scaffold to develop new antimicrobial agents. Thus, Simijonović et al. [62] synthesized two pyrogallol–coumarin-based hybrids, **9a**–**b**, displayed in Figure 13, and assessed their antimicrobial effect against thirteen antibacterial and three fungal strains, with tetracyclines and fluconazole used as reference drugs in vitro. Although the synthesized hybrids exhibited inferior results in comparison to reference drugs, they displayed a promising antibacterial effect, which was dependent on the type of bacterial strains used, with MMC and MIC values ranging between 0.8 and 2.70 mM. Notably, these hybrids exhibited better antibacterial activity against *P. mirabilis* ATCC 12,453 compared to other bacterial strains, with MMC and MIC of 0.17 and 0.08 mM, respectively. Additionally, hybrid **9b** displayed better antibacterial effects against several bacterial strains compared to **9a**. Moreover, when it comes to fungal strains, the hybrids exert promising antifungal activity against *C. albicans*, exhibiting MIC and MMC values between 0.17 and 1.35 mM, with hybrid **9a** displaying remarkable antifungal activity, as displayed in Table 9 [62]. The anti-biofilm activity results depicted that hybrid **9b** exhibited better anti-biofilm activity, especially against *S. aureus* ATCC 25923, with a BIC_50_ value of 1.02 mM, whereas hybrid **9a** showed no activity. This illustrates that hybrid **9b** was the most effective compound. However, more studies focused on aspects such as toxicology, in vivo, and the mechanism of action are recommended to supplement these findings [62].

**Figure 14:**
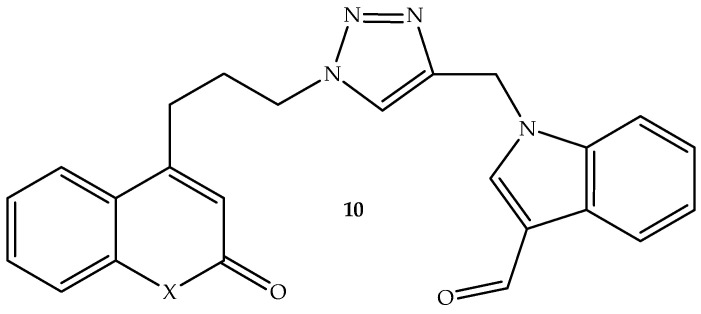
Chemical structure of indole–triazole-linked coumarin hybrid **10**.

An indole–triazole-linked coumarin hybrid **10**, shown in Figure 14, was synthesized by Khan et al. [63] and assessed against *S. aureus* and *E. coli* bacterial strains and *C. albicans* fungal strain in vitro, using tetracycline and fluconazole as reference drugs. Hybrid **10** displayed moderate antimicrobial activity against bacterial and fungal strains, with zone inhibition values (shown in Table 10) ranging from 10 to 16 mM, compared to 20 to 28 mM for the reference drugs, in a concentration-dependent manner. Notably, this compound showed more antibacterial activity against *S. aureus* compared to *E. coli* [63]. Hence, this compound’s mechanism of action was assessed on Thymidylate Kinase (PDB ID: 4QGG) and Dihydrofolate Reductase (PDB ID: 3SRQ) proteins, and it displayed better interaction with the amino acid residues via the hydrogen bonding, π-π stacking, and aromatic bonding, exhibiting docking scores of −9.657 kcal/mol and −9.102 kcal/mol compared to −7.270 kcal/mol and −8.259 kcal/mol of tetracycline. Therefore, this compound could be a potential replacement for tetracycline, as it binds better with the targeted proteins compared to tetracycline [63]. Hence, more studies are recommended to further assess this compound.

**Figure 15:**
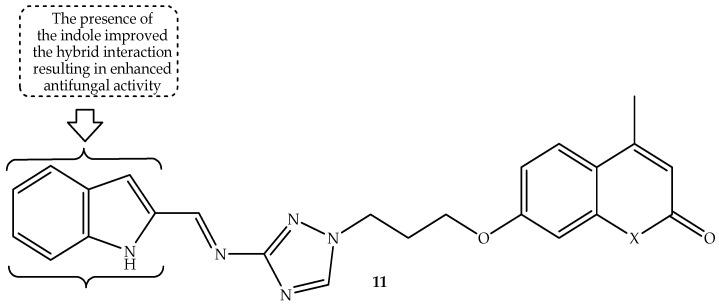
Chemical structure of triazole-linked coumarin hybrid **11**.

A series of triazole-linked coumarin hybrids were synthesized by Manda et al. [64] and evaluated against fungal strains, including *C. albicans*, *A. fumigatus*, and *A. niger*, with fluconazole and griseofulvin utilized as reference drugs. The antifungal findings displayed hybrid **11** (depicted in Figure 15) as the significant antifungal active hybrid among the synthesized compounds against *A. niger* and *C. albicans*, with MIC values of 31.25 µg/mL, which is higher than those of reference drugs, as depicted in Table 11 [64]. Consequently, this compound displays a good binding affinity towards the tubulin *α-β* heterodimer protein, showing H bonding, Van der Waals forces, and hydrophobic interactions with amino acid residues, exhibiting a docking score of −10.5 kcal/mol compared to −8.2 kcal/mol of griseofulvin. Notably, the type of substituents introduced in the synthesized hybrids influenced the antifungal activity of the hybrids, as hybrid **11** depicted that the presence of the indole in this hybrid improved its interaction with the amino acid residues, whereas its destruction resulted in compromised antifungal activity [64]. Overall, this hybrid showed remarkable antifungal activity, and it could be a potential lead facilitating the discovery of new antifungal agents. Hence, more studies, including Swiss ADMET, in vitro bacterial studies, and in vivo evaluations, are recommended for this compound.

**Figure 16:**
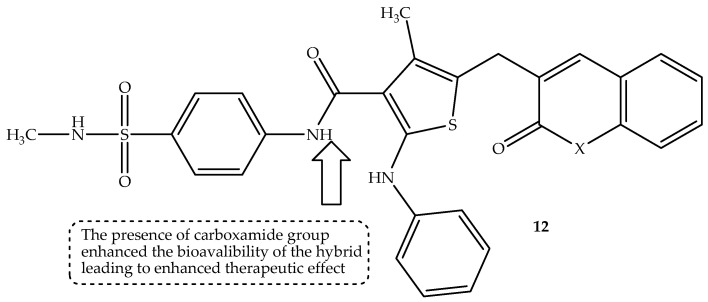
Chemical structure of thiophene–coumarin, **12**.

Abumelha et al. [65] synthesized a generation of thiophene–coumarin hybrids and evaluated them for their antimicrobial activity against four bacterial and two fungal strains, in vitro. The hybrids were developed by varying the functional groups attached to the third position of the thiophene moiety and replacing sulfonamide with carboxamide [65]. The hybrids displayed varied antimicrobial activity against the pathogens with MIC values comparable to those of ampicillin and flucytosine. Notably, hybrid **12** (displayed in Figure 16), displayed consistent promising antimicrobial activity among the synthesized compounds, with MIC values (displayed in Table 12) in a range of 38–144 µg/mL compared to 35–121 µg/mL of ampicillin against the bacterial strains and 88–117 µg/mL compared to 126–144 µg/mL of flucytosine against fungal strains, respectively. However, against *E. coli*, this compound displayed less activity compared to some hybrids [65]. The molecular docking findings revealed that the hybrids exhibited π-H and hydrogen bonding interactions with the amino acid residues, with hybrid **12** displaying the highest binding energy of −7.2776 kcal/mol against the 4URO protein [65]. Moreover, in silico Swiss ADME findings also corroborated the in vitro findings, displaying this compound as the most promising drug, as it exhibited improved pharmacokinetics with a harmonic balance in molecular weight, bioavailability, TPSA, and lipophilicity, despite violating Lipinski’s rule [65].

SAR displayed that the presence of 2-oxo-2H-chromene-3-carbonyl and thiophene moieties influenced the interaction of the hybrid with bacterial enzymes [65]. Furthermore, the phenylamino group enhanced the aromaticity and hydrogenation ability, leading to improved binding of the hybrids with specific targets in microbial cells. Moreover, the replacement of sulfonamide with the carboxamide group influenced the bioavailability and the solubility of the hybrids, leading to an improved antibacterial effect. Additionally, the presence of the *N*-methyl group positively contributed to the antibacterial activity of the hybrids, as it improved their lipophilic nature, and substituting the hydroxyl group with a methyl group also improved the hydrophobic nature of the hybrids, leading to better biological activity, as this improved the hybrids’ ability to cross the cell membranes [65]. In addition, the presence of the NH_2_ group, in contrast to hydroxyl and methyl groups, further improved the hydrogen bonding formation capabilities, resulting in improved interactions [65].

The DNA gyrase inhibition findings displayed that although the compounds displayed less inhibition compared to novobiocin, the sulfonamide-free terminal showed better DNA gyrase inhibition compared to the methyl-substituted sulfonamide, and the amide-bridged hybrids also displayed better DNA gyrase inhibition results compared to the diazo-bridged hybrids. This illustrates the importance of structural modification to the ability of the drug to bind with bacterial enzymes. Hence, the type of substituents and linkers must be considered when developing hybrid compounds. In essence, the synthesized thiophene–coumarin hybrids proved that structural modification could influence the antimicrobial activity of the compounds, as the compounds displayed promising antimicrobial activity. Therefore, more structural modifications are recommended for these hybrids to further improve their therapeutic effect [65].

**Figure 17:**
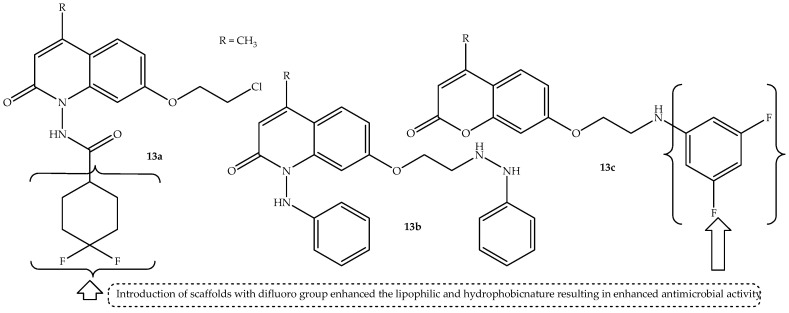
Chemical structure of quinolone–coumarin hybrid, **13a**–**c**.

A generation of quinolone–coumarin hybrids were synthesized using a microwave irradiation method by Lakshmi et al. [66] and colleagues, and they were biologically evaluated against different microbial strains in vitro. The hybrids displayed different bacterial effects against *E. coli*, *S. pyogenes*, *S. aureus*, and *P. aeruginosa*, with *E. coli* displaying more susceptibility to the hybrids. Additionally, against the antifungal strains, the compounds showed promising antifungal effect against both *C. albicans* and *A. niger*, displaying compromised antifungal activity against *A. fumigatus*. Notably, hybrid **13a**–**c** (depicted in Figure 17) displayed a significant antibacterial effect against *E. coli* compared to other synthesized hybrids and the standard drug (ciprofloxacin), with MIC values of 15.62 μg/mL compared to 31.25 μg/mL of ciprofloxacin, as shown in Table 13. These hybrids also displayed remarkable antifungal activity compared to their counterparts, showing MIC values of 31.25 μg/mL (except **13c** against *A. niger* with MIC = 62.25 μg/mL) compared to 62.5 μg/mL and 125 μg/mL of the standard drug (griseofulvin) against both *C. albicans* and *A. niger*, respectively [66].

Consequently, these hybrids displayed good binding interaction with the tubulin alpha beta heterodimer and peptide formylase receptor, with binding energies ranging between −8.8 and −9.4 and −7.7 and −8.5 compared to −7.6 and −7.1 of griseofulvin and ciprofloxacin, respectively. The molecular docking studies displayed that the hybrids bind better with the amino acid residues of the pathogens via π-cation, hydrogen bonding, π-alkyl, π-σ, π-π, and van der Waals forces interaction [66]. The SAR displayed that the introduction of difluorocyclohexanecarboxamide resulted in hybrid **13a** with enhanced lipophilicity, leading to improved antimicrobial activity. Similarly, attaching phenyl hydrazine to the first and seventh positions of the quinolone moiety resulted in hybrid **13b** with improved biological activity, with the incorporation of 2,4-difluoroaniline enhancing the antimicrobial activity of hybrid **13c**. In essence, the nature of substituents in these hybrids influenced their antimicrobial activity, with the introduction of scaffolds with difluoro groups increasing their lipophilic and hydrophobic nature, resulting in enhanced antimicrobial activity [66]. Therefore, these encouraging findings depict that these hybrids are potent antimicrobial agents. However, more studies, including in vivo findings, are recommended.

**Figure 18:**
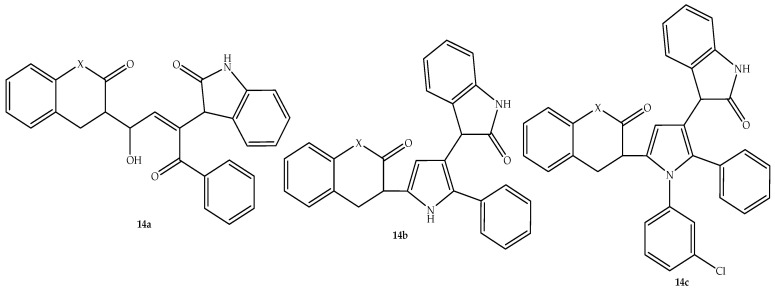
Chemical structure of isatin–coumarin-based hybrids, **14a**–**b**.

Allah et al. [67] synthesized isatin–coumarin-based hybrids as potent antimicrobial agents. The compounds were varied by introducing different scaffolds between the isatin and coumarin moieties. The hybrids exhibited different antibacterial activity, with inhibition zones (depicted in Table 14) ranging between 1 and 3 mm compared to 4–5 mm of the standard drugs against *B. subtilis*, *S. aureus*, and *E. coli*, in vitro [67]. Hybrids **14a**–**c** (depicted in Figure 18) displayed promising antibacterial activity compared to their counterparts, especially against Gram-positive bacterial strains. This illustrates the importance of *N*-(3-chlorophenyl) pyrrole and pyrazole, furan, and dibenzo[b,e][1,4]diazonin to the antimicrobial activity of these compounds, with hybrids without these moieties showing less activity [67]. Although these hybrids are displaying potential antibacterial activity, more studies, including in silico ADMET, molecular docking, and clinical trials, are recommended for these hybrid compounds to validate the current findings.

**Figure 19:**
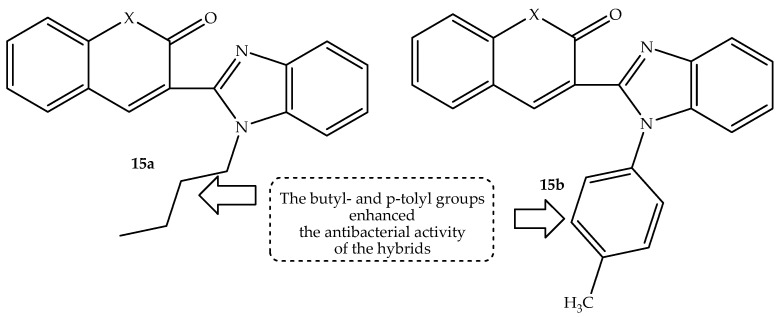
Chemical structure of coumarin–benzimidazole hybrids, **15a**–**b**.

A series of coumarin–benzimidazole hybrids with varied substituents at different positions of the benzimidazole moiety were developed by Arya et al. [68] and evaluated against both Gram-positive and Gram-negative bacterial strains, with vancomycin, meropenem, and polymyxin-B utilized as standard drugs. Hybrid **15a** (butyl-substituted) and **15b** (p-tolyl-substituted), shown in Figure 19, were the compounds with the most antibacterial activity among the synthesized compounds and vancomycin against the Gram-positive bacterial strain (*K. pneumoniae*) with MIC values of 6.25 µg/mL, as depicted in Table 15.

Notably, other synthesized compounds were inactive against all the strains [68]. Additionally, the cytotoxicity results indicated that these compounds showed better activity compared to the standard drugs at a lower concentration (50 µM). However, increasing the concentrations (>100 µM) decreased the normal tissue survival rates by 3-fold. Therefore, these compounds were further submitted for in vivo evaluations against *K. pneumoniae*-infected mice. The compounds significantly reduced the bacterial counts comparable to meropenem at increased doses. Hence, these compounds can be recommended as potential replacements for meropenem, and they could be a steppingstone towards new therapeutic agents for the treatment of *K. pneumoniae*-related infections. Thus, in silico, the compounds displayed good drug likeness and physicochemical properties, with only hybrid **15b** violating Lipinski’s rule. Moreover, the compounds display high BBB-penetrating ability, illustrating that they could be potent for the central nervous system infections. Furthermore, these two antibacterial compounds showed no carcinogenicity or mutagenicity. These findings confirm these two drugs as potential treatments for bacterial infections [68].

**Figure 20:**
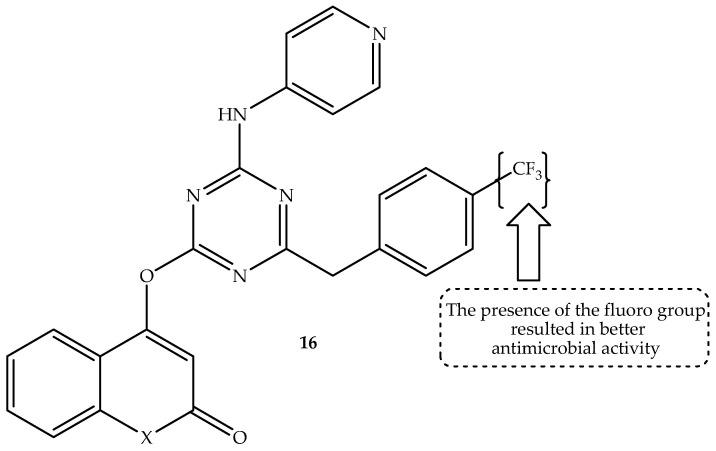
Chemical structure of coumarin–s-triazine-linked hybrids, **16**.

Zala et al. [69] hybridized pyridine, benzothiazole, and coumarin via s-triazine linker, resulting in novel potential antimicrobial candidates when tested against selected bacterial and fungal strains in vitro. Norfloxacin and griseofulvin were used as a reference drug. The hybrids displayed moderate to good antibacterial activity on different strains [69]. As displayed in Table 16, among the synthesized coumarin hybrids, compound **16** (displayed in Figure 20) was the most promising, with an MIC value of 25 µg/mL, 25 µg/mL, and 12.5 µg/mL compared to 10–50 µg/mL of norfloxacin against human *B. subtilis*, *C. tetani*, and *E. coli* strains, respectively. Consequently, hybrid 16 was also the most promising antifungal agent with comparable antifungal activity to griseofulvin, displaying MIC values of 100 µg/mL and 500 µg/mL against *T. rubrum* and *C. albicans* antifungal strains, respectively [69].

Moreover, this compound was submitted for in silico molecular docking on *S. aureus* dihydropteroate synthetase. It displayed a potent binding ability with *S. aureus* dihydropteroate synthetase, showing a binding score of −6.596 kcal/mol compared to −8.198 kcal/mol of the co-crystallized inhibitor. Notably, the presence of the electron-withdrawing group (CF_3_) was reported to improve the antimicrobial activity of this hybrid. Hence, it was the strongest antibacterial candidate among the synthesized coumarin hybrids [69]. However, more studies, such as in vivo studies, are recommended for these coumarin-based hybrids to supplement these findings.

**Figure 21:**
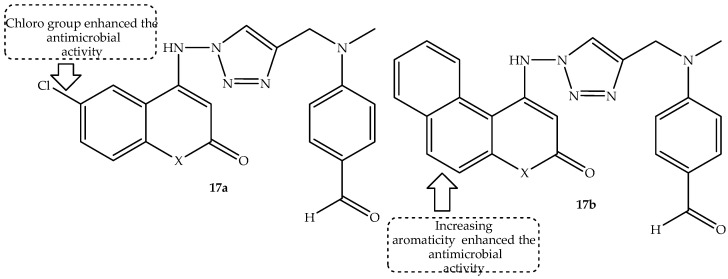
Chemical structure of coumarin–1,2,3-triazole hybrids, **17a**–**b**.

A generation of coumarin-1,2,3-triazole hybrids was synthesized through copper(I)-catalyzed azide alkyne cycloaddition by Jaggal and coworkers as potential antibacterial agents in comparison to streptomycin [70]. Among the synthesized hybrids, a marginal difference displayed hybrid **17a**–**b** (shown in Figure 21) as the most promising antibacterial active compounds, with MIC values between 0.5 and 4 μg/mL compared to 0.5–1 μg/mL of streptomycin against selected bacterial strains, including *S. aureus*, *S. pyogenes*, *S. typhi*, and *P. aeruginosa* (the MIC values are summarized in Table 17).

The SAR showed that the position and type of substituents present in these compounds influenced their antibacterial activity, with the halogenated **17a** (chloro group at position 6 of coumarin) and benzo-fused **17b** (5,6-benzo coumarin) displaying remarkable activity compared to their counterparts. The improved π-π interaction of these drugs was suspected to be responsible for the enhanced activity [70]. Hence, in silico molecular docking was conducted to test the interaction of these drugs with amino acid residues of *S. aureus* dihydropteroate synthase. These compounds showed good binding energies with binding scores between −8.4 kcal/mol to −9.6 kcal/mol via pi–anion, carbon–hydrogen bonding, π-π stacked, hydrogen bonding, pi–alkyl, and alkyl interactions. Additionally, hybrid **17a**–**b** displayed better pharmacokinetics and improved oral bioavailability due to higher intestinal absorption, in silico. Moreover, these hybrids displayed better tissue distribution, were unable to pass the BBB, and posed no toxicity threats to normal tissues. However, the compounds displayed some toxic traits as they inhibited Pg-substrates and CYP1A2, suggesting a possibility of drug–drug interactions. Therefore, further investigations, including in vivo evaluations, are recommended for these two antibacterial active hybrid compounds [70].

**Figure 22:**
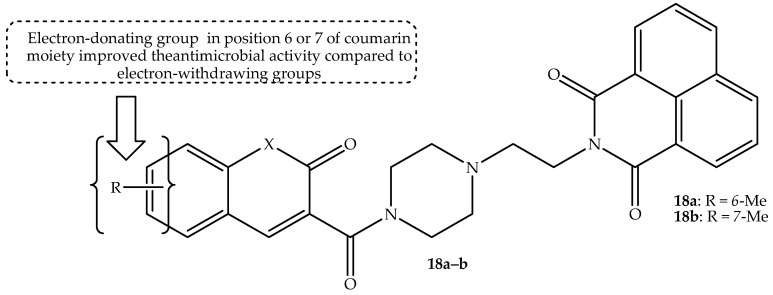
Chemical structure of coumarin–naphthalimide hybrids, **18a**–**b**.

The DNA intercalation and the pharmacological properties of naphthalimide prompted Rana et al. [71] to synthesize a generation of coumarin–naphthalimide hybrids with varied substituents at positions 6 and 7 of the coumarin and test them against selected bacterial strains. The coumarin–naphthalimide hybrids were modified via the introduction of aromatic rings on the coumarin moiety, varying the type of linkers and chain length between the two scaffolds, inserting the carbonyl group between the two moieties, and replacing the piperazine ring. Hybrids **18a** and **18b** (displayed in Figure 22) exhibited remarkable antibacterial activity, with MIC values ranging between 1 µg/mL and 0.5 µg/mL against *S. aureus*, respectively. Notably, most of the synthesized compounds displayed activity against *S. aureus* compared to other bacterial strains in vitro [71]. The SAR displayed that the presence of the electron-donating groups at positions 6 and 7 of the coumarin ring (such as in hybrids **18a**–**b**) was crucial for the antibacterial activity in comparison to the hybrids with electron-withdrawing groups. Additionally, replacing the piperazine ring with a propyl chain and aminopyridine linkers between the coumarin and naphthalimide moieties compromised the antibacterial activity of the compounds. Furthermore, changing the chain length with carbonyl groups, replacing coumarin with other heterocyclic scaffolds, and introducing substituents on the naphthalimide moiety also resulted in a loss of antibacterial activity [71]. This illustrates the importance of naphthalimide, coumarin, and piperazine linkers in the antibacterial activity of these hybrids. Moreover, hybrids (**18a**–**b**) were evaluated for cytotoxicity against Vero cell lines, and they were non-toxic with CC_50_ values of more than 50 µg/mL and a selective index of more than 12.5. The fractional inhibitory concentration (FIC) index, in comparison to the Food and Drug Administration (FDA)-approved drugs, displayed no antagonistic effect. Moreover, the in silico molecular docking studies revealed that hybrid **18a** binds with amino acid residues of *S. aureus* DNA gyrase via π-π stacking and hydrogen bonding interactions. Additionally, it was noted that hybrids **18a**–**b** are bacteriostatic, as they inhibit the topoisomerase IV enzymes [71]. Hence, more studies, including in vivo evaluation, are recommended for these two drugs.

**Figure 23:**
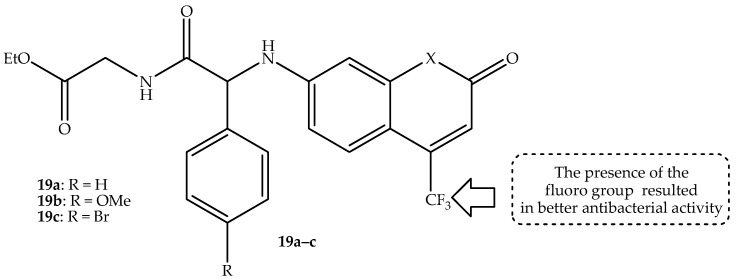
Chemical structures of trifluoromethyl-containing coumarin hybrids, **19a**–**c**.

Kowalczyk et al. [72] synthesized the trifluoromethyl-containing coumarin hybrids for the treatment of bacterial meningitis-related infections. In vitro, against several *E. coli* and *S. aureus*, the compounds displayed promising antibacterial activity with MIC and MBC values ranging from 0.58 to 1.25 µg/mL and 0.62–1.43 µg/mL, respectively. Notably, compounds containing the CF_3_ group, such as hybrid **19a**–**c** (depicted in Figure 23), exhibited more antibacterial activity compared to the methyl group-containing hybrids. Additionally, the compounds were more selective towards the *E. coli* compared to the *S. aureus* strains, and this was ascribed to the presence of lipopolysaccharide in the outer membrane of the *E. coli* strains [72]. The cytotoxicity tested against normal BALB/c-3T3 and αT3-1 cell lines revealed that the compounds showed no toxic threats towards normal tissues, with excellent cell viability percentages of more than 96% at low concentrations (0.5–1 µg/mL). This showcased these hybrids as potent bactericidal agents, as their cytotoxicity results were comparable or lower to the used FDA-approved antibiotics, including bleomycin, ciprofloxacin, and cloxacillin. Additionally, the in vivo evaluations of these coumarin hybrids were performed on the cerebrospinal fluid of a sheep to test their ability to pass the BBB [72]. Hybrid **19a** displayed outstanding ability to pass the BBB, as more concentrations were observed in the cerebrospinal fluid compared to its counterparts. This was attributed to its structural architecture, and the presence of CF_3_ further improved its ability to pass the BBB, as CF_3_ improves the lipophilic nature of the compounds and C-F bonds can inhibit metabolic degradation, leading to improved bioavailability. Therefore, hybrid **19a** can be recommended for the treatment of bacterial infections, especially in the central nervous system. However, more in vivo studies using a wide range of concentrations, instead of only one dose, were recommended to reinforce these findings [72]. Hence, further research on these components is still sorely needed.

**Figure 24:**
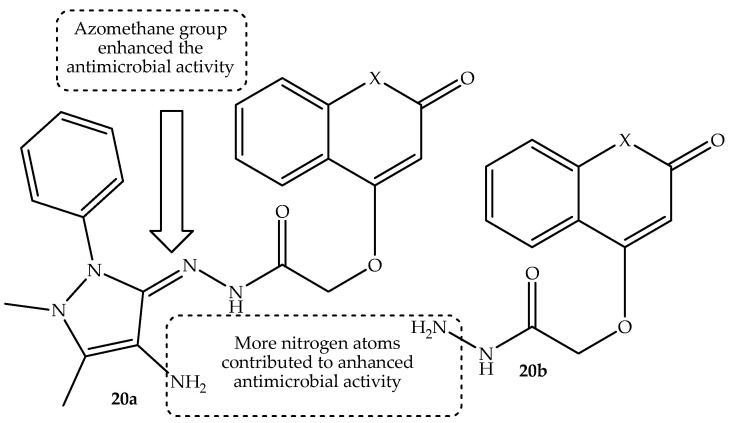
Chemical structure of coumarin hybrid, **20a**–**b**.

Another series of coumarin hybrids were synthesized by Betti et al. [73] and evaluated to determine their antimicrobial activity against selected bacterial and fungal strains. The synthesized hybrids displayed better antimicrobial activity compared to their parent compounds, and the presence of azomethane and nitrogen atoms incorporated in these hybrids positively influenced their antimicrobial effect [73]. Compound **20a** (shown in Figure 24) exhibited an outstanding antibacterial effect in a concentration-dependent manner against all used bacterial strains, especially *E. coli*. Additionally, the exceptional performance of hybrid **20a** was supplemented by the presence of the 4-aminoantipyrine moiety, which improved the stability of this hybrid. Furthermore, the lipophilic nature of these compounds was enhanced by the ability of pi electron delocalization, resulting in enhanced bacterial membrane permeability. Notably, hybrid **20b** (depicted in Figure 24) exhibited remarkable antifungal activity among the synthesized hybrids against *C. albicans* and *A. niger*, with the presence of aromatic ring and hydrazine moieties being responsible for the improved interaction with fungal cells. Thus, the proposed antibacterial and antifungal action of these hybrids involves disruption of protein synthesis, DNA replication, membrane distribution and disruption, induction and generation of reactive oxygen species, and protein denaturation. Therefore, hybrid **20a**–**b** were recommended as potent antimicrobial agents [73]. However, more studies are urgently needed to validate these findings and confirm these proposed mechanisms of action.

**Figure 25:**
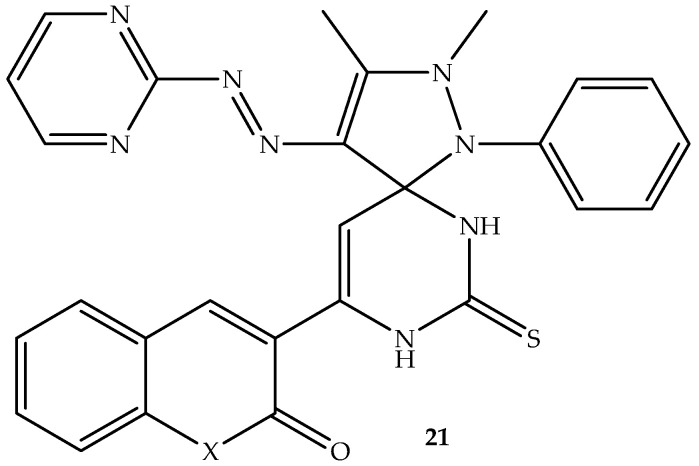
Chemical structure of spiro-heterocyclic coumarin hybrid, **21**.

A series of spiro-heterocyclic coumarin hybrids were synthesized by Al-Burgus et al. [74] and evaluated against *S. aureus* and *E. coli* bacterial strains. Among the five synthesized hybrids, compound **21** (depicted in Figure 25) displayed remarkable activity against both bacterial strains in vitro [74]. Hence, its ability to interact with DNA gyrase was studied in silico. The compound displayed better binding affinity with the DNA gyrase amino acid residues, displaying binding scores of −9.9 kcal/mol and −8.2 kcal/mol compared to −7.2 kcal/mol and −6.8 kcal/mol of clorobiocin against *E. coli* and *S. aureus*, respectively. The hybrids showed pi-anions, pi-alkyl, pi–sigma, amide-pi-stacked, and pi-cation interactions with amino acid residues via hydrogen bonding using lactones from the coumarin, pyrimidine, and thiol group. Although there are synthetic limitations, such as high costs, harsh conditions, and low yields of these spiro-heterocyclic coumarin hybrids, the findings revealed hybrid **21** to be a potential antibacterial candidate [74]. Therefore, more studies are recommended for this compound.

**Figure 26:**
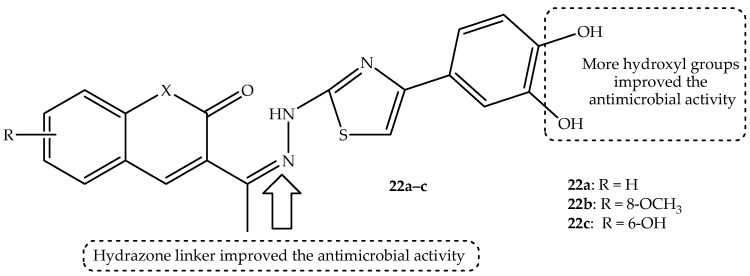
Chemical structures of 3,4-dihydroxyphenyl-thiazole-coumarin hybrids, **22a**–**c**.

Interesting findings were reported by Ungureanu et al. [75] regarding the antimicrobial efficacy of 3,4-dihydroxyphenyl–thiazole–coumarin hybrids. The hybrids exhibited promising antibacterial activity, with MIC and MBC values that were superior to and comparable to the control drugs, especially against *S. aureus* and *P. aeruginosa*, with *S. aureus* showing more susceptibility to the hybrids compared to *P. aeruginosa*. Hybrid **22a** showed remarkable antibacterial activity comparable to that of ciprofloxacin, with MIC and MBC values between 15.62 and 31.25 µg/mL against *E. coli*, *P. aeruginosa*, and *S. aureus* bacterial strains, as displayed in Table 18 [75]. The hybrids showed antifungal activity comparable with fluconazole against *C. albicans* and *A. brasiliensis*. Hybrids **22a**–**c** (shown in Figure 26) exhibited the most promising antifungal activity against the selected antifungal strains, with MIC and MFC values between 7.81 and 31.25 µg/mL and 15.62 and 31.25 µg/mL, respectively [75]. Notably, the synthesized hybrids were categorized as fungicidal and bactericidal as they displayed MIC/MBC and MIC/MBC ratios of less than four. Additionally, the SAR showed that introducing ether groups into position eight of coumarin compromised the antibacterial activity of the hybrids, with the presence of the hydrazone linker between coumarin and azomethine nitrogen moieties also influencing the antifungal activity.

Moreover, the presence of the hydroxyl group at position 7 of coumarin resulted in hybrid **22c** with enhanced overall antimicrobial activity. Thus, this hybrid also exhibited noticeable antibiofilm activity against *P. aeruginosa*; hence, it can be recommended as a promising candidate for the treatment of *P. aeruginosa*-related infections. The introduction of bromine and a hydroxyl group at position 6 and aromatic ring on the coumarin scaffolds positively influenced the antibiofilm activity of these hybrids. The hybrids displayed good binding affinities compared to the novobiocin and displayed steric interactions with the amino acid residues [75]. The in silico ADMET predictions revealed that hybrid **22c** does not display any drug–drug pharmacokinetics interaction threats. However, it is among the list of hybrids with no BBB penetration capacity and low GI absorption, leading to compromised oral administration. Additionally, this hybrid showed high toxicity in silico, as it belongs to class III for acute oral toxicity [75]. Hence, in vivo studies are recommended to reinforce these findings.

**Figure 27:**
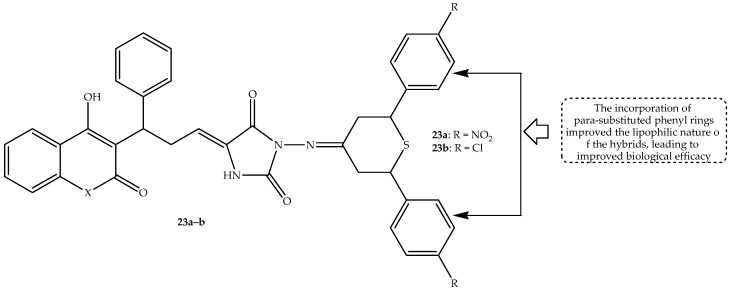
Chemical structure of coumarin-thiopyrans-imidazolidine hybrids, **23a**–**b**.

Loganathan et al. [76] used Knoevenagel condensation to synthesize a series of potential antimicrobial coumarin–thiopyrans–imidazolidine hybrids. The hybrids were tested against several fungal and bacterial strains, and they displayed a promising antimicrobial effect. The MIC values depicted in Table 19 reveal that hybrid **23a** (displayed in Figure 27) was the most antibacterial active compound among the synthesized hybrids, with an MIC value of 2 μg/mL compared to 4 μg/mL of ciprofloxacin against the resistant *S. aureus* bacterial strain. Moreover, hybrid **23b** (depicted in Figure 27) was the most antifungal active compound among the synthesized hybrids, with comparable MIC values between 0.5 and 1 μg/mL to clotrimazole against *C. albicans* and *A. niger* fungal strains [76]. Notably, the antimicrobial activity findings exposed that the combination of the coumarin and thiopyran–imidazolidine scaffolds resulted in enhanced biological activity, as the parent compounds exhibited far less antimicrobial activity compared to the hybrids. Additionally, the incorporation of para-substituted phenyl rings improved the biological activity of the drugs, as this improved the lipophilic nature of the hybrids. Therefore, hybrid **23a**–**b** can be potent antimicrobial agents [76]. Hence, more evaluations, such as in silico and in vivo studies, are recommended for these hybrids.

**Figure 28:**
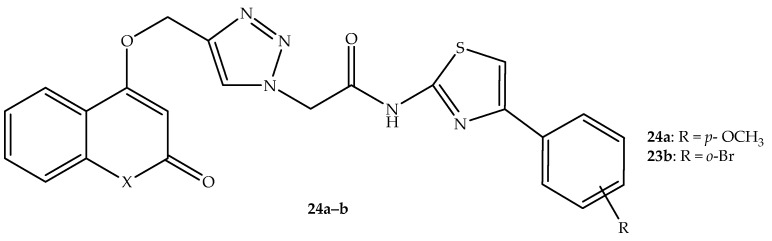
Chemical structure of coumarin–triazole hybrids, **24a**–**b**.

Click chemistry reactions were utilized by Kangad et al. [77] to synthesize potential antimicrobial coumarin–triazole hybrids. The hybrids exhibited moderate to good antimicrobial activity. However, the antimicrobial effect was inferior compared to that of ciprofloxacin, ampicillin, and nystatin [77]. Hybrids **24a**–**b** (depicted in Figure 28) exhibited more antibacterial activity with MIC values of 125 µg/mL against *E. coli*, *B. subtilis*, *S. aureus*, and *S. typhimurium* strains, as depicted in Table 20. The in silico molecular docking studies displayed that these compounds bind well with *C. albicans* CYP51 enzymes via hydrophobic interactions, with binding energies between −9.56 to −10. 13 kcal/mol. This illustrates that these hybrids can be a potent treatment for *C. albicans*-related fungal infections. However, further research was recommended for these hybrids [77].

## 6. Conclusions and Future Recommendations

The prevalence of microbial infections is a global priority, as it is evident that the number of people affected by microbial infections could escalate by 2050. Natural occurring heterocyclic compounds, such as coumarin, are reported to be a potential candidate for the development of a new treatment for microbial infections. However, challenges such as drug resistance and poor bioavailability limit their biological efficacy. Hence, coumarin is hybridized with other pharmacophores to develop new drugs for microbial infections. This review proves that coumarin contributes to drug discovery, as its presence contributes to the improved antimicrobial activity of the new drugs in silico, in vitro, and in vivo. Notably, the structural modification must be considered when hybridizing the coumarins with other pharmacophores, as the nature of substituents present in the compounds and the type of linkers used to combine the moieties are reported to affect the antimicrobial outcomes of the hybrids, as depicted in Table 21. For instance, the trend of the presence of aromatic rings, nitro, hydroxyl, chloro, bromo, and fluoro groups improves the lipophilic and hydrophobic nature of the compounds, resulting in improved membrane permeability and enhanced biological efficacy. Therefore, incorporating these types of functional groups at different positions of the substituted aromatic rings could result in new bactericidal and fungicidal hybrids.

Moreover, it is evident from molecular docking studies that coumarin hybrids commonly interact better with amino acid residues of the DNA gyrase via different interaction mechanisms, and it is noted that the mutated DNA gyrase commonly causes drug resistance. Therefore, these coumarin hybrids can be a potential treatment for bacterial infections, and the DNA gyrase can be the primary target for the coumarin-containing antibacterial compounds. Additionally, hybridizing coumarin with specific DNA gyrase inhibitors could result in more effective therapeutic agents for bacterial infections. Furthermore, *S. aureus* bacterial strains and *C. albicans* were reported to be more susceptible to the reported coumarin hybrids. This illustrates that coumarin hybrids must be considered more in the treatment of *S. aureus* and *C. albicans*-related infections. The reported literature on coumarin hybrids reinforces the importance of the in silico ADMET predictions in drug discovery, as the in silico predictions exposed vital properties of the new hybrids that collaborate with in vitro and in vivo findings in the reported literature. Hence, the in silico evaluation can be used as an optimization tool, as this approach could accelerate the process of moving from preclinical to clinical trials. Moreover, the incorporation of the hybrids into drug carriers could also reduce their toxicity to normal tissues, as coumarins are reported to cause hepatotoxicity at high doses. Hence, its specificity must be improved to enhance the biodistribution and bioavailability of these hybrids into the targeted sites.

## Figures and Tables

**Table 1:** In vitro antimicrobial findings of hybrid **1** in comparison to the reference drugs.

Compounds	Inhibition Zone (mm)				
	*S. aureus*	*E. coli*	*P. aeroginosa*	*S. pyogenes*	*C. albicans*
**1**	8	14	15	10	11
Ciprofloxacin	25	20	21	23	Not tested
Fluconazole	Not tested	Not tested	Not tested	Not tested	26

**Table 2:** In vitro antimicrobial findings of hybrid **2** in comparison to the reference drug.

Compounds	Inhibition Zone (mm)		MIC Values (µg/mL)	
	*S. aureus*	*C. albicans*	*S. aureus*	*C. albicans*
**2**	28	34	4.88	9.76
Neomycin	28	28	19.53	39.06

**Table 3:** In vitro antimicrobial findings of hybrids **3a**–**b** in comparison to the reference drug.

Compounds	Inhibition Zone (mm)	
	*E. coli*	*P. aeruginosa*
**3a**	12	<12
**3b**	<12	13
Ampicillin	25	25

**Table 4:** In vitro antimicrobial findings of hybrids **4a**–**b** in comparison to the reference drugs.

Compounds	Inhibition Zone (mm)		
	*S. aureus*	*P. aeruginosa*	*C. albicans*
**4a**	15.43	14.8	4.08
**4b**	10.51	5.93	10.43
Fluconazole	Not tested	Not tested	0.41
Colistin	Not tested	0.18	Not tested
Vancomycin	0.67	Not tested	Not tested

**Table 5:** In vitro antimicrobial findings of hybrids **5a**–**c** in comparison to the reference drugs.

Compounds	MIC Values (μg/mL)		
	*E. coli*	*P. aeruginosa*	*C. albicans*
**5a**	1	0.125	64
**5b**	0.5	0.25	256
**5c**	0.5	0.25	32
Ciprofloxacin	0.5	0.125	64
Cephalexin	0.5	0.25	256

**Table 6:** In vitro antimicrobial findings of hybrids **6a**–**d** in comparison to the reference drugs.

Microbial Strains	MIC Values (µg/mL)						
	6a	6b	6c	6d	Ciprofloxacin	Nystatin	Metformin
*E. coli*	4	2	16	8	1	Not tested	Not tested
*P. aeruginosa*	4	2	16	4	1	Not tested	Not tested
*S. dysenteriae*	4	2	16	4	0.5	Not tested	Not tested
*S. typhi*	4	2	16	8	1	Not tested	Not tested
*H. influenzae*	4	2	16	8	1	Not tested	Not tested
*K. pneumonia*	4	2	8	4	0.5	Not tested	Not tested
*B. fragilis*	9	29	48	30	Not tested	Not tested	3
*C. perfringens*	6	25	48	27	Not tested	Not tested	0.7
*F. necrophorum*	7	24	32	32	Not tested	Not tested	1.85
*P. melaninogenica*	9	32	44	36	Not tested	Not tested	0.8
*A. niger*	10	1.8	1.3	1.65	Not tested	8	Not tested
*C. albicans*	5	1.55	1.15	1.35	Not tested	4	Not tested

**Table 7:** Percentage inhibition growth (%) of hybrids **7a**–**b** in comparison to the reference drugs.

Compounds	Percentage Inhibition Growth (%)	
	*K. pneumonia*	*C. albicans*
**7a**	24.84	17.44
**7b**	20.20	33.47
Colistin	0.18	Not tested
Fluconazole	Not tested	0.41

**Table 8:** Inhibition Zone (mm) of hybrids **8a**–**b** in comparison to the reference drugs.

Compounds	Inhibition Zone (mm)				
	*S. aureus*	*P. aeruginosa*	*E. coli*	*S. pyogenes*	*C. albicans*
**8a**	16	17	10	16	20
**8b**	11	10	8	10	25
Fluconazole	Not tested	Not tested	Not tested	Not tested	30
Ciprofloxacin	18	Not tested	22	15	Not tested

**Table 9:** In vitro MIC values (mM) of hybrids **9a–b** in comparison to the reference drugs.

Microbial Strains	MIC Values (mM)		
	9a	9b	Tetracycline/Fluconazole
*S.boulardii*	1.35	2.70	0.025
*S. cerevisiae*	1.35	1.35	0.025
*C. albicans ATCC 10231*	0.17	0.17	0.1
*S. enterica*	0.17	0.08	0.001
*E. coli*	0.34	0.17	0.005
*P. aeruginosa ATCC 27853*	0.34	0.34	0.02
*P. mirabilis ATCC 12453*	0.08	0.08	0.035
*K. pneumoniae*	0.17	0.17	0.009
*L. plantarum 299V*	2.70	0.67	<0.001
*S. aureus*	2.70	1.35	<0.001
*S. aureus ATCC 25923*	0.17	0.08	<0.001
*B. subtilis*	1.35	1.35	0.002
*B. subtilis ATCC 6633*	2.70	0.34	0.005

**Table 10:** Zone inhibition values (mm) of hybrid **10** in comparison to the reference drugs.

Microbial Strains	Zone Inhibition Values (mm)		
	10	Tetracycline	Fluconazole
*E. coli*	10–12	24	Not tested
*S. aureus*	10–16	28	Not tested
*C. albicans*	12–15	Not tested	20

**Table 11:** In vitro MIC values (µg/mL) of hybrid **11** in comparison to the reference drugs.

Compounds	MIC Values (µg/mL)		
	*C. albicans*	*A. fumigatus*	*A. niger*
**11**	31.25	62.5	31.25
Griseofulvin	62.5	125	125
Fluconazole	62.5	125	31.25

**Table 12:** In vitro MIC values (µg/mL) of hybrid **12** in comparison to the reference drugs.

Compounds	MIC Values (µg/mL)					
	*E. coli*	*K. pneumoniae*	*S. aureus*	*S. epidermidis*	*A. fumigatus*	*C. albicans*
**12**	144	44	38	76	117	88
Ampicillin	121	35	50	61	Not tested	Not tested
Flucytosine	Not tested	Not tested	Not tested	Not tested	144	126

**Table 13:** In vitro MIC values (µg/mL) of hybrid **13a–c** in comparison to the reference drugs.

Compounds	MIC Values (µg/mL)						
	*E. coli*	*S. pyogenes*	*S. aureus*	*P. aeruginosa*	*A. fumigatus*	*C. albicans*	*A. niger*
**13a**	15.62	31.25	31.25	32.25	250	31.25	31.25
**13b**	15.62	15.62	125	31.25	125	31.25	31.25
**13c**	15.62	31.25	125	15.62	125	31.25	62.50
Ciprofloxacin	31.25	31.25	125	125	Not tested	Not tested	Not tested
Griseofulvin	Not tested	Not tested	Not tested	Not tested	62.50	62.50	125

**Table 14:** Inhibition zones (mm) of hybrid **14a**–**c** in comparison to the reference drugs.

Compounds	Inhibition Zone Values (mm)		
	*E. coli*	*B. subtilis*	*S. aureus*
**14a**	2	3	2
**14b**	2	3	3
**14c**	1	2	1
Streptomycin	5	5	4
Gentamycin	Not tested	4	Not tested
Penicillin G	Not tested	5	Not tested

**Table 15:** In vitro MIC values (µg/mL) of hybrid **15a**–**b** in comparison to the reference drug.

Compounds	MIC Values (µg/mL)		
	*K. pneumoniae*	*A. baumannii*	*P. aeruginosa*
**15a**	6.25	>100	>100
**15b**	6.25	>100	>100
Vancomycin	>50	12.5	>50

**Table 16:** In vitro MIC values (µg/mL) of hybrid **16** in comparison to the reference drugs.

Compounds	MIC Values (µg/mL)				
	*B. subtilis*	*C. tetani*	*E. coli*	*T. rubrum*	*C. albicans*
**16**	25	25	12.5	100	500
Norfloxacin	10	50	10	Not tested	Not tested
Vancomycin	Not tested	Not tested	Not tested	100	500

**Table 17:** In vitro MIC values (µg/mL) of hybrid **17a**–**b** in comparison to the reference drug.

Compounds	MIC Values (µg/mL)			
	*S. aureus*	*S. pyogenes*	*S. typhi*	*P. aeruginosa*
**17a**	1	2	4	2
**17b**	0.5	1	2	2
Streptomycin	0.5	0.5	1	2

**Table 18:** In vitro MIC values (µg/mL) of hybrids **22a**–**b** in comparison to the reference drugs.

Compounds	MIC Values (µg/mL)				
	*P. aeruginosa*	*S. aureus*	*E. coli*	*C. albicans*	*A. brasiliensis*
**22a**	31.25	15.62	15.62	15.62	31.25
**22b**	31.25	15.62	125	7.81	15.62
**22c**	31.25	16.52	62.5	15.62	31.25
Ciprofloxacin	31.25	15.62	15.62	Not tested	Not tested
Fluconazole	Not tested	Not tested	Not tested	15.62	>250

**Table 19:** In vitro MIC values (µg/mL) of hybrids **23a**–**b** in comparison to the reference drugs.

Compounds	MIC Values (µg/mL)		
	*S. aureus*	*A. niger*	*C. albicans*
**23a**	2	8	4
**23b**	4	1	0.5
Ciprofloxacin	4	Not tested	Not tested
Clotrimazole 1	Not tested	1	0.5

**Table 20:** In vitro MIC values (µg/mL) of hybrids **24a–b** in comparison to the reference drugs.

Compounds	MIC Values (µg/mL)			
	*S. aureus*	*B. subtilis*	*E. coli*	*S. typhimurium*
**24a**	500	500	125	125
**24b**	125	125	500	500
Ciprofloxacin	Not tested	Not tested	50	50
Ampicillin	100	100	Not tested	Not tested

**Table 21:** Synopsis of SAR and molecular docking targets of the documented hybrids, **1**–**24**.

Hybrids	SAR Findings	Docking Target	References
**1**	The nitro group exhibited more antimicrobial activity compared to the bromo, chloro, and methyl groups.	-	Farhan et al. [53]
**2**	NH of the pyridine ring, C-O-C, and C-O of the pyrone ring contributed to the antibacterial activity by improving the interaction with the targeted site.	DNA gyrase	Abo-Salem et al. [54]
**3a**–**b**	The halogenation in the ortho position of the chalcone and aromatic ring groups enhanced the activity by improving the hybrid interaction with the target site, with the ester linker and bromo group reported to further improve the lipophilicity of the hybrids, resulting in enhanced antibacterial effect.	-	Ngaini et al. [55]
**4a**–**b**	Mono-substituted hybrids with the most electron-withdrawing group, such as the nitro group, exhibited better antimicrobial activity compared to those with electron-donating groups.	-	Dhawan et al. [56]
**5a**–**c**	The introduction of different substituents on the pyran ring influenced the antimicrobial activity, with hybrids containing pyran-pyrazole displayed better antifungal activity compared to those containing pyran-pyrimidines.	DNA gyrase	Fayed et al. [57]
**6a**–**c**	The presence of the dioxathiole ring and introduction of different substituents in the para position of the phenyl ring moiety had an impact on the hydrophilic nature of the compounds, following this order: F < Cl < OCH_3_ < CH_3_ < Br < I.	-	Zeki et al. [58]
**7a**–**b**	Halogenation (chloro and bromo) in the ortho position of the phenyl ring moiety improved the antimicrobial activity.	-	Dhawan et al. [59]
**10**	No obvious trends	Thymidylate Kinase and Dihydrofolate Reductase proteins	Khan et al. [63]
**11**	The presence of the indole in this hybrid improved its interaction, resulting in enhanced antifungal activity.	tubulin *α-β* heterodimer protein	Manda et al. [64]
**12**	The presence of different substituents improved the hydrophobic nature and binding interaction of the hybrids, leading to improved antimicrobial activity in the following hierarchy: OH < CH_3_ < NH_2_. Moreover, the carboxamide group improved the bioavailability of the hybrids compared to the sulfonamide group.	DNA gyrase	Abumelha et al. [65]
**13a**–**c**	Introduction of difluorocyclohexanecarboxamide, attaching phenyl hydrazine to the first and seventh position of the quinolone moiety, and incorporation of 2,4-difluoroaniline, enhanced the antimicrobial activity of the hybrids. The incorporation of difluoro groups increases their lipophilic and hydrophobic nature, leading to improved activity.	tubulin alpha beta heterodimer and peptide formylase receptor	Lakshmi et al. [66]
**14a**–**b**	The presence of *N*-(3-chlorophenyl) pyrrole and pyrazole, furan, and dibenzo[b,e][1,4]diazonin enhanced the antimicrobial activity of the hybrids.	-	Allah et al. [67]
**15a**–**b**	The butyl- and p-tolyl groups enhanced the antibacterial activity of the hybrids.	-	Arya et al. [68]
**16a**–**b**	The presence of the fluoro group (CF_3_) resulted in better antimicrobial activity.	Dihydropteroate synthetase	Zala et al. [69]
**17a**–**b**	The halogenation (chloro) and enhanced aromaticity improved the antibacterial effect of the hybrids	Dihydropteroate synthase	Jaggal et al. [70]
**18a**–**b**	The electron-donating groups at positions 6 and 7 were more preferable than electron-withdrawing groups, with the replacement of the piperazine ring resulting in compromised antibacterial activity. Additionally, replacing alkyl chain linkers with carbonyl linkers resulted in a loss of the activity.	DNA gyrase and topoisomerase IV enzymes	Rana et al. [71]
**19a**–**c**	The presence of the fluoro group (CF_3_) resulted in better antibacterial activity.	-	Kowalczyk et al. [72]
**20a**–**b**	The presence of azomethane and nitrogen atoms positively influenced the antimicrobial effect of the hybrids. Moreover, the 4-aminoantipyrine moiety was responsible for improving the stability of the hybrids, enhancing the antibacterial effect, with the presence of the hydrazine moiety and aromatic ring resulting in hybrids with improved antifungal activity.	-	Betti et al. [73]
**21**	Coumarin, pyrimidine, and thiol moieties improved the interaction of the compounds with amino acid residues.	DNA gyrase	Al-Burgus et al. [74]
**22a**–**c**	Introduction of ether groups comprised the antimicrobial activity of the hybrids, and the use of a hydrazone linker and hydroxyl group positively influenced the activity. Additionally, the introduction of aromatic rings, hydroxyl, and bromo groups improved the antibiofilm activity, leading to enhanced overall antimicrobial activity.	GyrB subunit and CYP51 enzymes	Ungureanu et al. [75]
**23a**–**b**	The incorporation of para-substituted phenyl rings improved the lipophilic nature of the hybrids, leading to improved biological efficacy.	-	Loganathan et al. [76]
**24a**–**b**	-	CYP51 enzymes	Kangad et al. [77]

## Data Availability

Not applicable.

## Abbreviations

The following abbreviations are used in this manuscript:

CNS	Central Nervous System.
FDC	Fixed-Dose Therapy.
SAR	Structural Relationship Activity.
GI	Gastrointestinal.
PT	Polytherapy.
ADMET	Absorption, Distribution, Metabolism, Excretion, and Toxicity.
DNA	Deoxyribonucleic acid.
MIC	Minimum Inhibitory Concentration.
MFC	Minimum Fungicidal Concentration.
MBC	Minimum Bactericidal Concentration.
FIC	Fractional Inhibitory Concentration.
FDA	Food and Drug Administration.
TPSA	Topological Polar Surface Area.
BBB	Blood-Brain-Barrier
